# Emerging Role of Carotid MRI for Personalized Ischemic Stroke Risk Prediction in Patients With Carotid Artery Stenosis

**DOI:** 10.3389/fneur.2021.718438

**Published:** 2021-08-03

**Authors:** Kelly P. H. Nies, Luc J. M. Smits, Mohamed Kassem, Paul J. Nederkoorn, Robert J. van Oostenbrugge, M. Eline Kooi

**Affiliations:** ^1^Department of Radiology, CARIM School for Cardiovascular Diseases, Maastricht University, Maastricht, Netherlands; ^2^Department of Radiology and Nuclear Medicine, Maastricht University Medical Center, Maastricht, Netherlands; ^3^Department of Epidemiology, Maastricht University, Maastricht, Netherlands; ^4^Department of Neurology, Amsterdam University Medical Center, Amsterdam, Netherlands; ^5^Department of Neurology, Maastricht University Medical Center, Maastricht, Netherlands

**Keywords:** ICA stenosis, ischemic stroke, TIA, prediction, vulnerable plaque, MRI

## Abstract

Rupture of a vulnerable carotid plaque is an important cause of ischemic stroke. Prediction models can support medical decision-making by estimating individual probabilities of future events, while magnetic resonance imaging (MRI) can provide detailed information on plaque vulnerability. In this review, prediction models for medium to long-term (>90 days) prediction of recurrent ischemic stroke among patients on best medical treatment for carotid stenosis are evaluated, and the emerging role of MRI of the carotid plaque for personalized ischemic stroke prediction is discussed. A systematic search identified two models; the European Carotid Surgery Trial (ECST) medical model, and the Symptomatic Carotid Atheroma Inflammation Lumen stenosis (SCAIL) score. We critically appraised these models by means of criteria derived from the CHARMS (CHecklist for critical Appraisal and data extraction for systematic Reviews of prediction Modeling Studies) and PROBAST (Prediction model Risk Of Bias ASsessment Tool). We found both models to be at high risk of bias. The ECST model, the most widely used model, was derived from data of large but relatively old trials (1980s and 1990s), not reflecting lower risks of ischemic stroke resulting from improvements in drug treatment (e.g., statins and anti-platelet therapy). The SCAIL model, based on the degree of stenosis and positron emission tomography/computed tomography (PET/CT)-based plaque inflammation, was derived and externally validated in limited samples. Clinical implementation of the SCAIL model can be challenging due to high costs and low accessibility of PET/CT. MRI is a more readily available, lower-cost modality that has been extensively validated to visualize all the hallmarks of plaque vulnerability. The MRI methods to identify the different plaque features are described. Intraplaque hemorrhage (IPH), a lipid-rich necrotic core (LRNC), and a thin or ruptured fibrous cap (TRFC) on MRI have shown to strongly predict stroke in meta-analyses. To improve personalized risk prediction, carotid plaque features should be included in prediction models. Prediction of stroke in patients with carotid stenosis needs modernization, and carotid MRI has potential in providing strong predictors for that goal.

## Introduction

Stroke is the second leading cause of death and the second largest contributor to global disability-adjusted life years (DALYs) since 2015 (1). Around 15% of all acute ischemic strokes are associated with extracranial carotid stenosis due to atherosclerosis (2). While currently a trend of a decreasing incidence of ischemic stroke is seen as a result of improved management of cardiovascular disease, projections made for European countries show that within 30 years the total number of ischemic strokes will increase by around 13% due to demographic changes (3, 4). The management of individuals at risk of stroke will need to be further improved to reduce the disease burden.

Current guidelines for patients with carotid artery stenosis distinguish them into two categories: patients to be treated only by best medical therapy, and patients eligible for additional surgical intervention by Carotid Endarterectomy (CEA), alternatively, Carotid Arterial Stenting (CAS).

Medical decisions are to a large degree dependable upon the degree of stenosis as well as other important risk factors such as clinical symptoms, age, and sex (5). In general, the benefit of performing CEA is seen in the group of recently symptomatic patients with a degree of stenosis of 70–99% and is considered in symptomatic male patients with 50–69% carotid stenosis (6). However, for symptomatic patients with 50–69% stenosis, the number needed to treat (NNT) to prevent one recurrent ischemic stroke is relatively high (NNT:15) (5, 7).

For patients with an asymptomatic 50–99% carotid stenosis, the risk of an ipsilateral ischemic stroke could now, due to improvements in best medical therapy, be lower than 1% per annum (8). Reported procedural risks of ischemic stroke and death when performing CEA measured after 2005, are 2.68% (95% CI, 2.12–3.31) and 1.50% (95% CI, 1.01–2.07) in symptomatic and asymptomatic patients, respectively (9). This can imply that, in some patients, revascularization causes more harm than benefit (10). In particular, in the group of symptomatic patients with 50–69% stenosis or asymptomatic patients, physicians may want to take additional risk factors (apart from the degree of stenosis) into consideration when making treatment decisions.

Results from the European Carotid Surgery Trial (ECST) have shown that almost half of symptomatic patients had a degree of stenosis <30% (11). Other factors must therefore be considered to improve risk stratification. Ischemic stroke caused by carotid artery disease is typically the result of embolization after carotid plaque rupture (12). An inflammatory response is triggered by the accumulation of oxidized low-density lipoprotein (LDL) in the arterial intima potentially leading to foam cell formation (13). Apoptosis and necrosis of the foam cells leads to the build-up of a lipid-rich necrotic core (LRNC). Plaque neovessels support the entry of more monocytes into the plaque, however, these vessels are fragile, which could cause intraplaque hemorrhage (IPH) (14). Also fissures or disruption of the fibrous cap (FC) may contribute to the development of IPH (15). The FC is separating the lumen from the thrombogenic content of the plaque. Therefore a thin or ruptured fibrous cap (TRFC) contributes, together with IPH and a LRNC, to an increase in probability of plaque rupture (13). Plaque rupture releases the contents of the plaque which can lead to thrombus formation, embolization, downstream arterial occlusion, and subsequent stroke (16).

Non-invasive modalities to visualize the carotid plaque are ultrasonography, computed tomography (CT), positron emission tomography (PET), and magnetic resonance imaging (MRI) (17–20). Ultrasonography and CT are unable to reliably differentiate the LRNC from IPH. PET provides information on inflammation in the plaque, but not on plaque composition. MRI is able to distinguish clearly between different soft tissues and is the only modality that enables the assessment of the presence of IPH, one of the most important vulnerable plaque features (12). MRI can facilitate the measurement of all the hallmarks of plaque vulnerability by using multiple different high spatial resolution contrast weightings and it is extensively validated to identify plaque burden, IPH, ulcerations, LRNC, and TRFC (12, 21).

Risk prediction models can help clinicians in weighing risks and benefits of treatment decisions. A risk prediction model is a mathematical equation that uses patient risk factor information as an input to estimate the probability of the patient having the health outcome of interest, now or in the future. The most widely used model for calculating the risk of ischemic stroke in symptomatic patients with carotid stenosis is the ECST medical score, which includes, besides the severity of stenosis, several additional risk factors such as hypertension, diabetes, and ulceration of the plaque (22). Besides the ECST medical model, recently another ischemic stroke risk prediction model has been developed and validated in symptomatic patients with carotid stenosis, the SCAIL-score, that is based on degree of stenosis and plaque inflammation as quantified with ^18^F-FDG PET-CT (23). However, other than ulceration and inflammation, features of plaque vulnerability have not been included in any prediction model for the risk of ischemic stroke in patients with carotid stenosis to date. Although some characteristics have in the meantime shown to be of high prognostic value for the occurrence of new or recurrent ischemic stroke with an even 10-fold increase in ischemic stroke in symptomatic patients with IPH on carotid MRI (24).

Since the development of the ECST medical score, novel MRI techniques to visualize the different components of the atherosclerotic carotid plaque have become available, and could improve prediction of individual ischemic stroke risk. In this review, we will systematically appraise the existing prognostic prediction models for the medium to long-term (≥90 days) risk of ischemic stroke in patients with carotid stenosis. In addition, we will discuss the potential additional predictive value of several MRI-based plaque features.

## Overview of Prediction Models

We performed a literature search in Pubmed to identify prediction models for medium to long-term (≥90 days) ischemic stroke risk in patients with medically managed carotid stenosis. The following search string was used in January 2021 to identify publications of interest; [(“Risk score^*^” or “Prediction model^*^” or “predictive model^*^” or “prognostic model^*^”)] AND (Carotid) AND (Stroke^*^ OR Transient Ischemic Attack^*^ OR TIA^*^). In total 265 results were evaluated and exclusion was based on: (1) not developed and/or validated in patients with carotid stenosis, (2) non-ischemic stroke as outcome, and (3) short term risk prediction (<90 days). This resulted in 11 articles of interest and in total two different predictive models both for patients with symptomatic carotid stenosis, while no models for asymptomatic patients could be identified within our search criteria. Additional publications on these predictive models were tracked using the article's list of references and articles citing the publication of interest. The final selection of articles was critically appraised by means of a data extraction and methodological assessment form based on the CHARMS (CHecklist for critical Appraisal and data extraction for systematic Reviews of prediction Modeling Studies) and PROBAST (Prediction model Risk Of Bias ASsessment Tool) criteria (25, 26). The different models were assessed for their general features, development, validation, performance, and feasibility in clinical practice by two assessors (KN and LS). Conflicts were resolved through joint discussion.

Based on CHARMS and PROBAST criteria, the ECST medical model, and symptomatic carotid atheroma inflammation lumen stenosis (SCAIL)-score both presented a high risk of bias. The derivation of the ECST medical model was of good quality, however clinical data based on trials from the 80s and 90s were used that no longer represent current ischemic stroke risks on best medical treatment and overall performance statistics were lacking. The SCAIL model has a high risk of bias due to a.o. inadequate reporting of derivation methods and insufficient derivation and validation sample size, especially when correcting for a range of clinical parameters. The findings are summarized in Table 1, and more elaborately discussed below.

**Table 1:** Overview and assessment of prediction models of recurrent acute ischemic stroke in patients with carotid stenosis.

	**ECST medical model (6, 22)**	**SCAIL (23)**
Model characteristics	• 11 predictors • Target group: patients with TIA/ischemic stroke and 50–99% stenosis • Prediction horizon: 5 years • Outcome: ipsilateral ischemic stroke • Method: Cox proportional hazards	• 2 predictors (or 9 predictors by correction for clinical parameters) • Target group: patients with TIA/ minor ischemic stroke and 50–99% stenosis • Prediction horizon: 90 days • Outcome: ipsilateral ischemic stroke • Method: Cox proportional hazards
Development	• Derivation population: symptomatic patients (ischemic stroke/TIA) with 50–99% stenosis + EPV ~ 12 - Handling of missing values not reported - Derivation data no longer reflecting ischemic stroke risk with current best medical treatment ± Simplified risk scores	• Derivation population: symptomatic patients (minor ischemic stroke/TIA) with ≥50% stenosis - EPV < 2 (*n* of candidate predictors unclear) - No censoring of patients with CEA ± Simplified risk scores
Validation	• Validation population: Symptomatic patients (TIA or ischemic stroke) with 50–99% stenosis - No internal validation - Validation by same authors in same paper	• Validation population: Symptomatic patients (minor ischemic stroke/TIA) with ≥50% stenosis - Low number of events - 9-factor model was used - Validation by same authors in same paper
Performance	+ Good calibration - No C-statistic given - No sensitivity or specificity reported	+ High C-statistic - Unclear what the performance of the 2-predictor model is
Feasibility	+ Web-based calculator available - No disclaimer and no access to explanatory texts on website	+ Only 2 predictors (without correction for clinical parameters) - Low face validity - PET/CT is expensive and patients are exposed to ionizing radiation
Overall risk of bias	High risk of bias - Data collection prior to current best medical treatment - No clear performance indicators	High risk of bias - Very low EPV - Validation performed with low number of events - Long-term prognostic power for patients with carotid stenosis not yet clear

### ECST Medical Model

The ECST medical model was first established in 1999 by Rothwell et al. on the basis of data of symptomatic patients with 0–69% carotid stenosis in the ECST (6). The degree of stenosis was determined with ECST criteria, and the following predictors were selected: cerebral vs. ocular events, plaque surface irregularity, any events within the past 2 months, and carotid stenosis (per 10% increase). During the development of the first version of the model, the study was split in two groups; one of patients with 0–69% carotid stenosis used for derivation, and one of patients with ≥70% stenosis used for external validation. In order to validate the study in a population from different hospitals, the data was later transformed to match the ECST to the NASCET method for determining the degree of stenosis (27). Where the ECST method uses the estimated position of the vessel wall at the site of the stenosis in the denominator, the NASCET method uses the distal normal lumen diameter, which results in different degrees of stenosis (28). With the newly determined degree of NASCET stenosis, the model was re-derived in patients with 50–99% NASCET stenosis. As a result other predictors were selected in this second version of the model [predictors: stenosis (per 10%), near occlusion, male sex, age (per 10 years), time since last event (per 7 days), presenting event (ocular, single TIA, multiple TIAs, minor ischemic stroke, major ischemic stroke), diabetes, previous myocardial infarction, peripheral vascular disease, treated hypertension, irregular/ulcerated plaque]. The resulting number of selected variables was much larger than in the 1999 version. Assuming that the same candidate predictors were used as in the original development in 1999, the model would have in total 17 candidate predictors and two additional degrees of freedom due to categorization. Considering there were 227 events in the dataset, the events per variable (EPV) was approximately 12, above the generally suggested minimum of 10 EPV (29).

The ECST data on which the model was based, were gathered during 1981–1991 with follow-up extending until 1998. Since then and more specifically from the early 2000s onwards, drug treatment has changed rigorously, with a >60% increase in statin use within a time period of 12 years and an increase in anti-platelet use (30). The use of statins causes a relative risk reduction of 21% for stroke, while anti-platelet is associated with a 12% risk reduction of serious vascular events (31). Because of this, the ECST model may over-estimate the risk of ischemic stroke. The authors also didn't report the full model, since the intercept was not given.

Internal validation was not performed or reported on. External validation was performed using data from the North American Symptomatic Carotid Endarterectomy Trail (NASCET). Calibration appeared good. A calibration plot showed agreement between predicted and observed medical risk. C-statistic, sensitivity, and specificity, were not reported. Derivation and external validation was reported in the same publication. As the authors note themselves, models often perform less effective in an independent sample when they are validated by researchers other than those who constructed the model (22).

The ECST-score has been simplified into color-coded risk tables to increase usability and counteract overfitting with the disadvantage that this results in a loss of accuracy to some extent, since hazard ratios (HRs) calculated at two decimal level are rounded to whole numbers. However, this way of presentation is understandable in the context of the facilities at hand at the time the model was developed. The prediction model is also available online (www.stroke.ox.ac.uk). Explanatory texts provided in a link on the webpage are not accessible to everyone visiting the site, which hinders careful consideration of the model for clinicians using this webpage. Overall, even with good derivation methods, the model has a high risk of bias according to PROBAST principles, mainly due to incomplete reporting and development in an outdated dataset, and not due to methods of development.

### Symptomatic Carotid Atheroma Inflammation Lumen Stenosis

A recently published model for the estimation of the risk of recurrent ischemic stroke included 18F-flueorodeoxyglucose (18F-FDG) standardized uptake values on positron emission tomography-computed tomography (PET-CT) as a parameter for plaque inflammation. This model, i.e., SCAIL categorizes 18F-FDG uptake into four different SUVmax ranges with increasing risk points. The basic version of this model included only two predictors; 18F-FDG uptake and the degree of NASCET stenosis categorized in the ranges <50, 50–69, and >70%. Inclusion criteria of the derivation cohort included a carotid stenosis of >50%, however some patients originally classified as moderate stenosis were re-measured and re-classified with a stenosis between 30 and 49% and remained included. In total 109 patients with previous non-severe ischemic stroke or TIA in the previous 30 days were used for derivation. While 37 recurrent ischemic strokes occurred in this dataset, only eight were after the PET-CT examination, therefore only these events should be included. Notably, after deriving an alternative model using only those eight events, the authors corrected for several clinical risk factors including; age, sex, hypertension, diabetes mellitus, smoking, antiplatelet, and statin treatment in the model, thereby considerably decreasing the study's already low number of EPV, and increasing the risk of overfitting. This effectively changed the two-predictor model to a nine-predictor model.

Validation was performed in a cohort from two centers with in total 87 patients with a previous TIA or minor ischemic strokes with a maximum time period between index event and inclusion of 14 days (no mean presented). However, carotid revascularization was performed in 44% and it is not clear if these patients were censored at the time of surgery. In the validation study it is also not specified if PET-CT imaging was performed before or after recurrent ischemic stroke. Based on the model that included only the eight events occurring after PET-CT imaging, model performance, as expressed by the C-statistic was 0.82 (95% CI, 0.66–0.97) in the derivation cohort.

External validation resulted in a performance of 0.77 (95% CI, 0.67–0.87) at 90 days. Pooling of the derivation and validation studies was used to determine the sensitivity and specificity of the model. The scores were categorized into low (0–1), medium (2–3), and high (4–5) risk of recurrent ischemic stroke. Only 9% of patients could be categorized as low risk, and those with medium risk still had 18% risk of recurrent ischemic stroke (time frame unreported). Dependent on the score threshold of >3 or >4, sensitivity was 81 and 38% and specificity was 54 and 90%, respectively (23). Overall, the model was appraised at high risk of bias, mainly due to low EPV and the small validation cohort.

### Overall Considerations of Current Models

Both models were assessed at high risk of bias according to PROBAST guidelines, hindering justification of their use in medical practice. The ECST medical model appears to have good calibration in the population used for validation. Limitations are that proof of sensitivity and specificity and overall predictive power in conventionally used C-statistics and the receiver operating characteristic (ROC) was not provided, therefore the discriminative ability is not clear. A strength of this study is a good EPV made possible by the large scale of the study. However, ischemic stroke risks have decreased significantly since data collection making the model outdated for use in current medical practice.

The SCAIL model demonstrates the potential of using plaque vulnerability features in a risk model. With only two predictors, its predictive capacity is remarkable. However, the model is prone to overfitting because of the low EPV. In addition, the model performance is only reported combined with a correction for a large range of clinical risk factors, which actually transforms the model into a multi-factor model.

If future larger validation studies provide proof of performance without adjustment by other clinical parameters, the model faces other issues in terms of clinical implementation, because of cost-effectiveness and availability of the imaging modality. PET-CT is costly with a factor two higher costs compared to MRI and there are less PET-CT scanners available compared to MRI (32). Besides this, there are insufficient events in the derivation dataset to correct for clinical risk factors, therefore the 2-predictor model should be used to minimize the effect of overfitting. Consequently, the model may lack validity since the clinicians could hesitate using a model with only two parameters while other parameters have been shown to be predictive of recurrent stroke as well. The model also categorizes the majority of patients in a median risk profile where considerable risk of recurrent ischemic stroke still occurs. Consequently, there is a low probability of the model being implemented in clinical practice.

MRI may provide a more accessible and cost-effective method to measure vulnerable plaque features for inclusion in risk stratification. There is a need for a modernized prediction model of current risk of ischemic stroke. MRI-measured vulnerable plaque features have shown value as independent predictors and their inclusion in prediction models could provide improved identification of individuals categorized at high risk of ischemic stroke.

## Carotid MR Imaging

**Figure 1:**
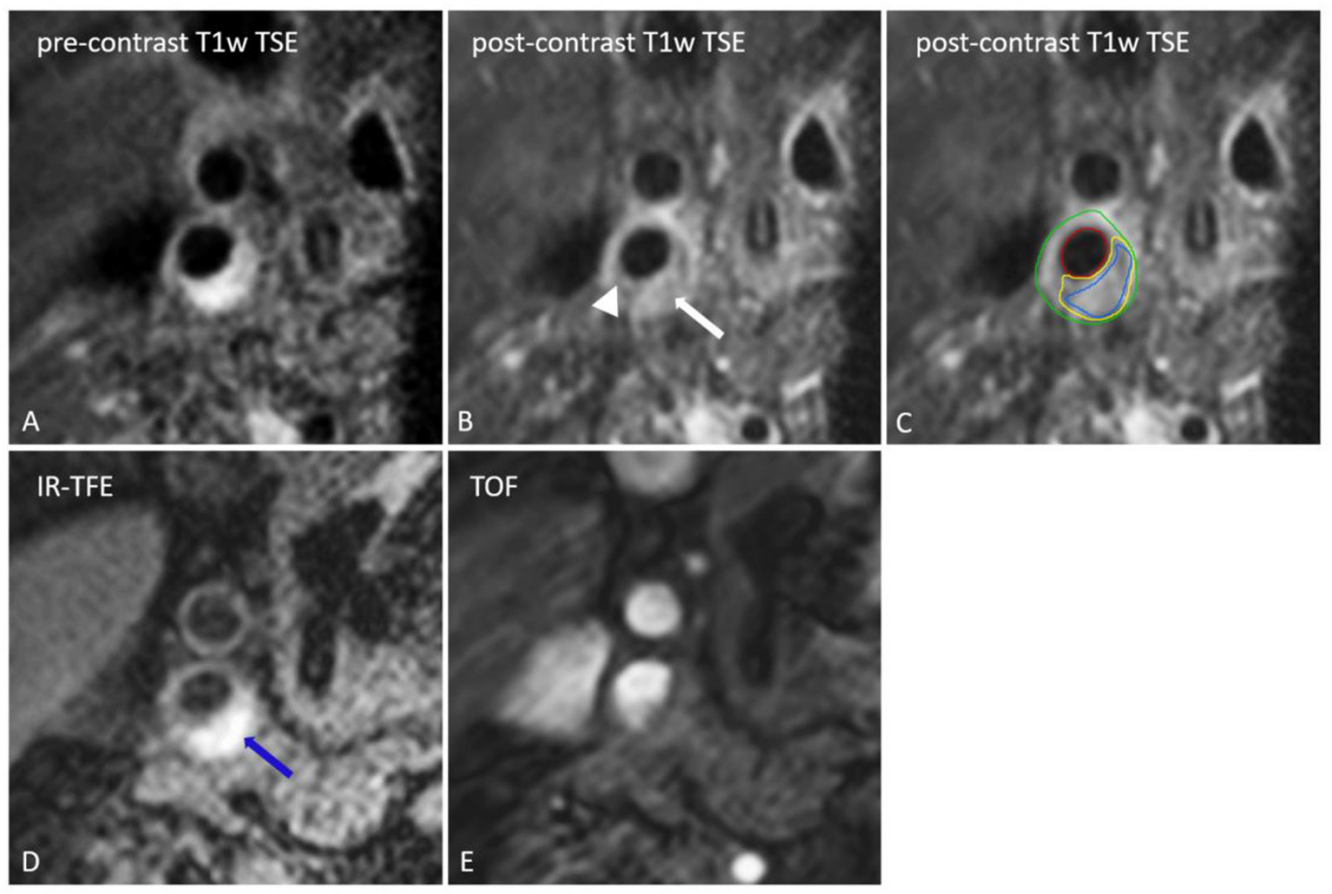
Transversal MR images of the right internal carotid artery. The black blood pre-contrast image **(A)** is used to draw the contours of the lumen and outer vessel wall. The lipid-rich necrotic core shows no contrast-enhancement on the post-contrast black-blood T1w quadruple inversion recovery (QIR) turbo spin echo (TSE) image **(B)** and includes the entire area of hemorrhage (IPH) [IPH: blue, lipid-rich necrotic core: yellow, lumen: red, outer vessel wall: green on **(C)**]. IPH [blue arrow on **(D)**] appears as a bright signal on the inversion recovery turbo field echo images (IR-TFE; **D**). A thin or ruptured fibrous cap (TRFC) can be identified by the interruption of juxtaluminal signal enhancement on the post-contrast T1w image (arrow head). With a contra-indication for contrast injection the T2w image or time of flight (TOF) image **(E)** can be used for TRFC assessment.

**Figure 2:**
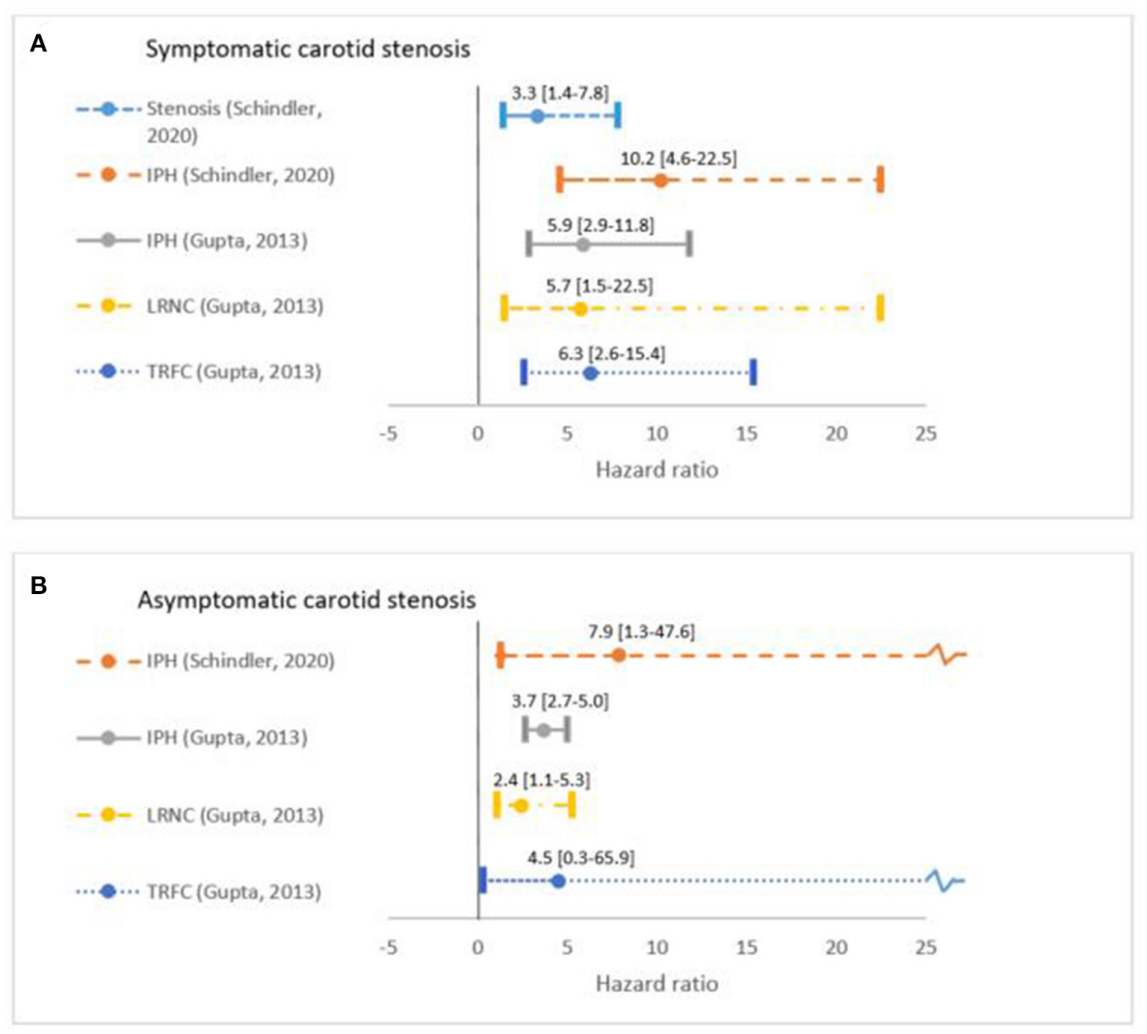
Overview of predictive value in Hazard Ratio [95% confidence interval] of plaque vulnerable features as reported in two meta-analyses. Schindler et al. (24) included 560 symptomatic and 136 asymptomatic participants with 66 ipsilateral ischemic strokes gathered from seven studies. The meta-analysis by Gupta et al. (37) consists of in total 779 patients (ratio symptomatic and asymptomatic unclear) with at least 169 ipsilateral ischemic strokes and TIAs (exact number unclear). Plaque volume is not included in this overview due to the lack of reported predictive value in meta-analyses. Patients are grouped into **(A)** symptomatic and **(B)** asymptomatic when data was available and hazard ratios were reported for the degree of stenosis, intraplaque hemorrhage (IPH), thin or ruptured fibrous cap (TRFC), and lipid-rich necrotic core (LRNC). Hazard ratios reported in Gupta et al. (37) include both ischemic stroke and TIA, while Schindler provided ischemic stroke hazard ratios.

Several imaging biomarkers have been suggested to provide insight into plaque vulnerability (18, 33). A vulnerable plaque is defined as a plaque that is prone to rupture. It is characterized by the presence of a large LRNC that is separated from the lumen by a TRFC. Upon rupture the blood gets in contact with the thrombogenic plaque content, which can cause thrombosis, embolization, and consequently, ischemic stroke (34). MRI is established as the most suited imaging technique to evaluate plaque composition, with its superior ability to differentiate between soft tissues (Figure 1) (35). Expert recommendations on carotid vessel wall MRI protocols have been published (18). For high resolution MR imaging, dedicated carotid radiofrequency coils are required, although IPH can be detected using a standard multi-channel neurovascular coil (18, 36). First, MRI methods to identify the different plaque features will be described. Next, the predictive value of the different plaque features were gathered from two large meta-analyses and will be discussed below (Figure 2).

### Degree of Stenosis

Generally, moderate stenosis is categorized as 50–69% stenosis, while the degree of stenosis is considered to be severe for 70% and above (38). The best non-invasive method for measuring the degrees of stenosis 70–90% is contrast-enhanced (CE)-MRA with a sensitivity and specificity of 0.94 (95% CI 0.88–0.97) and 0.93 (95% CI 0.89–0.96), respectively (Figure 1). Compared to the sensitivity and specificity of ultrasound and CTA, MRI performs significantly better in the different stenosis categories (38). For 50–69% of stenosis, MRI sensitivity is lower compared to higher degrees of stenosis, i.e., 0.77 (95% CI 0.59–0.89), while specificity remains very high (38). Time of flight (TOF)-MRA is not recommended because turbulent flow of recirculating blood can lead to underestimation of the degree of stenosis (39).

### Plaque Volume

Plaque volume can be determined by drawing manual or (semi-)automated contours delineating the outer- and inner-vessel wall on T_1_-weighted black-blood images. Pre-pulses are used to suppress the signal of blood to prevent plaque-mimicking artifacts (35). To account for changes in lumen size and wall thickness, the normalized wall index (NWI) is used as a reliable and reproducible method for calculating the percentage of wall area in total vessel area (40).

In response to an increase in atherosclerotic plaque volume, the artery may enlarge to allow enough luminal area for blood flow, which means that plaques could already be present without causing stenosis (41). An increase in plaque volume is also associated with a decrease of FC thickness and an increase of lipid proportion of the total plaque, further indicating its involvement in plaque vulnerability (41). Plaque progression is shown to be an independent predictor of recurrent ischemic stroke. Annual progression of carotid plaque volume in symptomatic patients (30–69% stenosis) was associated with an increased chance of recurrent ischemic stroke (HR: 1.19 per 10 mm^3^; 95% CI 1.03–1.37) (42). However, since this was determined in a relatively small study (63 patients, nine ischemic strokes), the need for larger trials to further assess the predictive value of plaque progression is needed.

### Intraplaque Hemorrhage

MRI is the only method that allows to accurately assess IPH presence in the carotid plaque. IPH can be recognized as a hyperintense signal in the bulk of a plaque in a hyper T1-weighted MR image, because of the methemoglobin shortening the T_1_-relaxation time (43). Magnetization-prepared rapid acquisition gradient (MP-RAGE), also referred to as inversion recovery turbo field echo (IR-TFE), is the most common sequence to visualize IPH presence with a high specificity (97%) and sensitivity (80%) compared to histology (44). Magnetization-prepared rapid acquisition gradient is able to suppress plaque components other than IPH with inversion-recovery preparation, allowing a clear differentiation between IPH, other plaque components and the lumen (45). Alternatively, 3D Simultaneous Non-contrast Angiography and IPH (3D-SNAP) has been developed to image stenosis and IPH using a single sequence (45). Other new developments include Multicontrast ATherosclerosis Characterization (MATCH), which simultaneously acquires hyper T1w, gray blood, and T2w images to visualize IPH, LRNC, and calcifications with a single 5 min sequence (46). Further clinical validation of these new sequences is needed.

IPH contributes to plaque vulnerability by causing an enlargement of the necrotic core size (47). IPH is out of the available plaque MRI predictors the most extensively validated and was shown to be a strong and independent predictor for ischemic stroke (24). Schindler et al. performed a meta-analysis with data pooled from seven cohort studies including 696 patients and reported an unadjusted ipsilateral ischemic stroke HR of 10.2 (95% confidence interval [CI]: 4.6–22.5) in symptomatic patients with vs. without IPH, and a HR of 7.9 (95% CI: 1.3–47.6) in asymptomatic patients. After adjusting for confounders, IPH remained significant and was identified as a strong independent ischemic stroke predictor (24). They also showed that the HR for severe degree of stenosis of 70–99% vs. <50% stenosis in symptomatic individuals was lower compared to IPH, i.e., 3.3 (95% CI: 1.4–7.8) (Figure 2).

At present, approximately 30% of ischemic stroke are categorized as cryptogenic because of a degree of stenosis <50%, however in some of these patients plaque rupture may also be the underlying cause of stroke, since Schindler et al. have demonstrated that in patients with <50% stenosis and IPH, ischemic stroke risk is increased from 0.7 to 9.0% with a mean follow-up of 18 months (24, 48, 49).

### Lipid-Rich Necrotic Core and Thin or Ruptured Fibrous Cap

Both the LRNC and the overlying FC can be visualized by comparing pre- and post-contrast T_1_ weighted black blood images, where the LRNC is the region within the bulk of the plaque that shows no or hardly no contrast enhancement, while a TRFC is identified as an interruption or absence of contrast enhancement in the juxtaluminal tissue overlying the LRNC (35). In case of a contraindication for contrast injection, a hypointensive signal on T_2_ weighted images is indicative for a LRNC, but it is sub-optimal to detect the LRNC because of an approximately two-fold lower signal-to-noise ratio (18).

Advanced plaques are characterized by a large LRNC separated from the lumen by a FC (12). A TRFC and presence of a LRNC increase the risk of ischemic cerebrovascular events by almost 6- and 3-fold, respectively, as reported by Gupta et al. from a clustered group of symptomatic and asymptomatic patients (Figure 2) (37, 50). A TRFC is also strongly associated with the presence of IPH (51).

## Discussion

A systematic search of prediction models for medium to long term risk of ischemic stroke resulted in the identification of two models for symptomatic carotid stenosis, and no models for asymptomatic carotid stenosis. Current prediction models, and in particular the ECST medical model, have provided clinicians with guidance in the selection of treatment based on the patients' risk of ischemic stroke. In clinical practice, its use accounts especially for borderline cases. We have appraised the prediction models according to CHARMS and PROBAST principles and were unable to find all crucial information on aspects of development, validation, and performance. While for both models claims are made of good performance after external validation, it should be noted that while the ECST medical model was validated in a good independent dataset and calibration appeared good, performance in terms of discrimination was not reported. SCAIL did report performance measurements, however the validation dataset was too small for accurate assessment. SCAIL has included more parameters than advised according to guidelines, resulting in increased chance of overfitting and potential loss of usability in different datasets other than the derivation data. Categorization of data and/or simplification of the model into risk scores was performed in both models to increase ease of use in clinical practice, however this decreases the accuracy of a model and when the model is presented in a web-based or an app-based approach simplification would not be needed. This was done for the ECST model with a web-based approach (www.stroke.ox.ac.uk) that has the potential for convenient implementation in clinical workflow.

The ECST-model provides good face validity and is therefore recommended in some national guidelines (52). However, this model is based on outdated patient data since treatment regime has changed dramatically in the last decades. The SCAIL model provides an interesting approach with only two parameters, when not correcting for other clinical risk factors, however development was performed in a very small sample size and due to the requirement of an additional of PET-CT examination it may struggle in face validity and feasibility in clinical practice. This model does show the great potential for using carotid plaque imaging for risk stratification models.

The use of carotid imaging of the plaque vulnerability in prediction models has not been fully exploited. Magnetic resonance imaging is currently the most promising imaging modality which can visualize the hallmarks of plaque vulnerability. For both symptomatic and asymptomatic patients, vulnerable plaque features on MRI showed strong associations with an increased risk of ischemic stroke. With a 10-fold increase in risk of ischemic stroke when IPH was present, or a HR close to six for the TRFC, the inclusion of these factors in a newly derived prediction model is expected to present greater predictive power. For any new prediction model, it would be important to use recent patient data, preferably collected after 2010 since best medical treatment was then last subject to vigorous changes by increased statin and anti-platelet use.

Ultimately, cost-effectiveness will play an important role in the adoption of new models in clinical practice. Feasibility of inclusion of certain plaque features in clinical practice will need to be reviewed by consultation of experts, analysis of costs associated with extra measurements, and the impact on the burden of disease.

In conclusion, current ischemic stroke risk prediction models for patients with symptomatic carotid artery disease have a high risk of bias, whereas there are currently no models to estimate the risk of ischemic stroke in asymptomatic carotid stenosis. There is an urgent medical need for modernized predictive models based on data from recent trials with the inclusion of newly identified carotid vessel wall imaging-based predictive factors. Carotid MRI biomarkers of plaque vulnerability, especially IPH, are most promising for this purpose.

## Author Contributions

KN, LS, and MKo contributed to the design of the study. KN and LS performed the analysis. KN wrote the first draft of the manuscript. MKa wrote sections of the manuscript. PN and RO provided feedback on intellectual content. All authors contributed to manuscript revision, read, and approved the submitted version.

## Conflict of Interest

The authors declare that the research was conducted in the absence of any commercial or financial relationships that could be construed as a potential conflict of interest.

## Publisher's Note

All claims expressed in this article are solely those of the authors and do not necessarily represent those of their affiliated organizations, or those of the publisher, the editors and the reviewers. Any product that may be evaluated in this article, or claim that may be made by its manufacturer, is not guaranteed or endorsed by the publisher.
